# Expectant management and live birth outcomes for male balanced-translocation carriers: Two case reports and a literature review

**DOI:** 10.1097/MD.0000000000020894

**Published:** 2020-06-26

**Authors:** Haitao Fan, Xiuyan Wang, Xiao Yang, Hongshu Zheng, Shuqiang Feng

**Affiliations:** Department of Urology, The Second Hospital of Jilin University, Changchun, China.

**Keywords:** balanced translocation, expectant management, live birth, recurrent pregnancy loss

## Abstract

**Rationale:**

Couples with male balanced-translocation carriers may experience recurrent pregnancy loss (RPL). Although the expectant management of RPL has developed over many years, genetic counseling for RPL couples with male balanced-translocation carriers remains challenging. Here, we describe the expectant management of 2 male carriers of balanced translocations.

**Patient concerns:**

A 32-year-old and a 28-year-old man presented at the clinic with diagnoses of infertility following spontaneous abortions by their wives.

**Diagnosis:**

Both patients had normal semen diagnosed by routine semen analysis and underwent cytogenetic diagnoses.

**Interventions:**

Following genetic counseling and informed consent, both couples voluntarily chose expectant management with natural conception.

**Outcomes:**

One couple experienced 2 natural pregnancies, the first of which ended in spontaneous abortion and the second produced a phenotypically normal infant. The other couple's first pregnancy resulted in a fetus with a balanced translocation confirmed by amniocentesis and cytogenetic analysis.

**Lessons:**

Expectant management with natural conception may be an alternative to genetic counseling in male balanced-translocation carriers with RPL, especially those who are reluctant to undergo preimplantation diagnosis.

## 1 Introduction

Chromosomal translocation is an important cause of genetic changes in humans.^[1]^ Reciprocal translocations are the most common structural rearrangement in infertile men.^[2]^ Male translocation carriers often exhibit reproductive issues such as male infertility or recurrent pregnancy loss (RPL) in their spouses.^[3]^ RPL is an obstetric complication that affects reproductive couples,^[4]^ and examination for possible chromosomal translocation in the male partner is essential.^[5]^ However, genetic counseling of these carriers remains challenging.^[6]^

Large studies have suggested 2 options for male reciprocal-translocation carriers who experience RPL. First, preimplantation genetic diagnosis (PGD) is recommended as a tool to improve live birth rates and reduce the rate of miscarriage.^[7]^ Fischer et al^[8]^ reported that translocation carriers who experienced 3 or more losses benefited from PGD, with an increased pregnancy-success rate, reduced length of time to conceive, and reduced pregnancy-loss rate. Scriven et al^[9]^ reported that PGD could benefit translocation carriers by reducing the risk of miscarriage and avoiding a pregnancy with an unbalanced form of the translocation. Second, further attempts at natural conception are also a viable option.^[10]^ Sugiura-Ogasawara et al^[11]^ reported that 63.0% of translocation carriers experienced a live birth following natural conception, and the cumulative live-birth rate with natural conception was reported to be 65% to 83%.^[12]^ Expectant management with natural conception has recently received attention for translocation carriers with RPL.

In clinical practice, parental carriers of chromosomal translocations and a history of RPL are more likely to pursue a natural pregnancy.^[13]^ Although PGD can reduce the abortion rate, it has a cost disadvantage.^[12]^ Furthermore, previous studies have shown no difference in reproductive outcomes, miscarriage rates, time to live birth, or live-birth rates between couples undergoing PGD and those pursuing a natural pregnancy.^[12–15]^ Hence, counseling for RPL patients with reciprocal translocation should include natural pregnancy or expectant management as treatment options.

This case report assesses the expectant management of 2 couples including men with chromosomal translocations, and further reviews its clinical application.

## 2 Case presentation

In these 2 cases, we carried out clinical expectant management for carriers of balanced translocations in couples with recurrent spontaneous abortions. This report was approved by the Ethics Committee of the Second Hospital, Jilin University, and written informed consent was obtained from both patients.

### 2.1 Case 1

**Figure 1:**
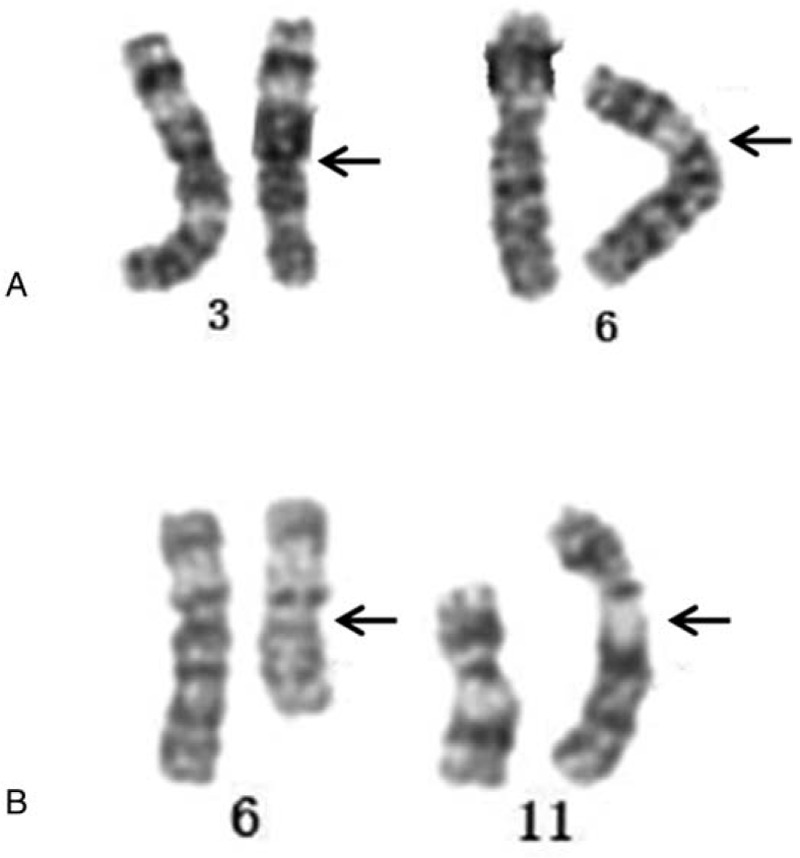
Abnormal partial karyotypes indicating chromosomal translocations.

A 32-year-old man visited the andrology service in March 2017 because his wife had experienced 2 spontaneous abortions before 13 weeks of gestation after 4 years of marriage. The patient had normal appearance and intelligence. Semen analysis revealed normal sperm concentration, motility, and morphology. The patient underwent cytogenetic detection, which revealed a karyotype of 46,XY,t(3;6)(q23;p21.3) (Fig. 1A). His wife's karyotype was 46,XX. Following genetic counseling, the couple refused PGD because of family and financial conditions, and chose to pursue natural conception. We considered expectant management as a possible option, and the couple provided informed consent for expectant management treatment. To improve the couple's confidence, we reassured them that balanced-translocation carriers can have natural pregnancies and produce phenotypically normal children. The couple received further attention before and during pregnancy, but the first pregnancy unfortunately resulted in spontaneous abortion at 10 weeks of gestation. However, a second pregnancy after 1 year passed 13 weeks of gestation successfully. Amniocentesis performed at 18 weeks of gestation showed that the fetus was a balanced-translocation carrier, consistent with the father's karyotype. A phenotypically normal child was subsequently born.

### 2.2 Case 2

An apparently normal 28-year-old man presented in April 2018 with a 3-year history of primary infertility, after his wife had experienced 3 spontaneous abortions before 13 weeks of gestation after 3 years of marriage. The patient had normal appearance and intelligence. Semen analysis revealed normal semen quality. The cytogenetic results revealed his karyotype as 46,XY,t(6;11)(q21;q25) (Fig. 1B). His wife's karyotype was 46,XX. Following genetic counseling, the couple provided informed consent for expectant management treatment. Their first attempted natural pregnancy passed 13 weeks safely, and amniocentesis and cytogenetic analysis at 17 weeks of gestation showed a fetus with a balanced translocation. This infant was subsequently delivered successfully.

### 2.3 Literature review

We searched for reports on expectant management of patients with chromosome translocations and RPL in PubMed using the keywords “expectant management/recurrent pregnancy loss.” Cases of chromosomal translocation were collected. A total of 11 studies involving live births following natural pregnancies were found. The reproductive outcomes of expectant management and natural conception in couples with recurrent abortion reported in previous studies are shown in Table 1. The live-birth rate for couples following natural conception was 25% to 71%.

**Table 1:**
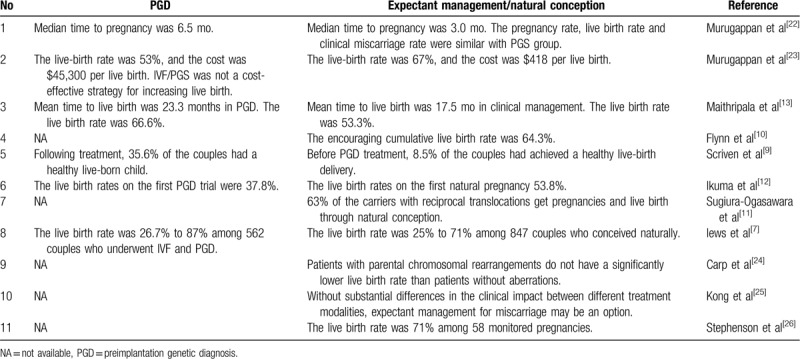
Reproductive outcomes of expectant management or natural conception for the couples with recurrent abortion reported in previous literature.

## 3 Discussion

In this study, we report the expectant management outcomes in 2 cases of RPL in couples with male balanced-chromosomal translocation carriers. One male carrier had the reciprocal translocation 46,XY,t(3;6) (q23;p21.3) and the other was 46,XY,t(6;11)(q21;q25). Parental chromosome rearrangements are one of the main causes of RPL, and the rates of pregnancy loss are higher in carriers compared with those with RPL and normal karyotypes.^[16]^ Balanced reciprocal translocations are the most common structural rearrangement.^[4]^ Men affected by such translocations may have failure of spermatogenesis and/or RPL.^[17]^ It has been reported that specific chromosomes and breakpoints involved in translocation are related to RPL,^[2,17,18]^ and the involved chromosomes and breakpoints should be considered in genetic counseling.

However, pregnancy resulting in live birth is possible in translocation carriers with RPL without treatment. Page et al^[16]^ reported that the rate of live births in carriers with balanced translocations was much higher than anticipated, with a live-birth rate of about 60% to 70% without treatment, depending on the specific chromosome or breakpoint.^[16]^ In addition, the pregnancy, live-birth, and clinical miscarriage rates were similar in patients treated with expectant management and those treated with PGD. Expectant management also has the benefit of lower cost per live birth, and expectant management is thus a worthy clinical option for balanced-translocation carriers.

Despite developments in expectant management, there are still challenges in relation to translocation carriers with RPL. This study explored the expectant management of 2 male carriers with RPL, involving chromosomes 3, 6, and 11 and breakpoints 3q23, 6p21.3, 6q21, and 11q25. Live births have previously been reported for male carriers with chromosome t(3,6)(q12;q27).^[19]^ In addition, familial balanced translocation carriers with t(3;6)(q12;q15) or t(3;6)(p12.3;q24.3) have frequently been reported, suggesting that their reproductive function is normal.^[20,21]^ Natural conception may be a more viable option for fertile carriers of translocations with a low risk of conceiving a chromosomally unbalanced offspring.^[9]^ To the best of our knowledge, this is the first report of the expectant management of carriers of chromosomal translocations with RPL in the Chinese population.

## 4 Conclusions

In conclusion, this study reported 2 couples including male balanced-translocation carriers who received expectant management. Natural conception with expectant management may be alternatives to genetic counseling in these patients, especially those who are reluctant to undergo PGD.

## Acknowledgment

The authors thank Susan Furness, PhD, from Liwen Bianji, Edanz Editing China (www.liwenbianji.cn/ac), for editing the English text of a draft of this manuscript.

## Author contributions

**Conceptualization:** Haitao Fan.

**Investigation:** Xiuyan Wang, Xiao Yang.

**Methodology:** Hongshu Zheng, Shuqiang Feng.

**Writing – original draft:** Xiuyan Wang.

**Writing – review & editing:** Haitao Fan.
